# Modified Keystone Perforator Island Flap for Tension-Reducing Coverage of Axillary Defects Secondary to Radical Excision of Chronic Inflammatory Skin Lesions: A Retrospective Case Series

**DOI:** 10.1155/2022/5600450

**Published:** 2022-09-29

**Authors:** Keun Hyung Kim, Byung Woo Yoo, Soo Yeon Lim, Kap Sung Oh, Junekyu Kim, Hyun Woo Shin, Kyu Nam Kim

**Affiliations:** ^1^Department of Plastic and Reconstructive Surgery, Konyang University Hospital, University of Konyang, College of Medicine, Myunggok Medical Research Center, 685 Gasuwon-dong seo-gu, Daejeon 35365, Republic of Korea; ^2^Department of Plastic and Reconstructive Surgery, Kangbuk Samsung Hospital, Sungkyunkwan University School of Medicine, 29, Saemunan-ro, Jongno-gu, Seoul 03181, Republic of Korea

## Abstract

Axillary defect coverage is often challenging after radical excision of chronic inflammatory skin lesions, such as complicated epidermoid cysts and hidradenitis suppurativa. This retrospective case series aims to demonstrate our experience with axillary reconstruction using the modified keystone perforator island flap (KPIF) technique, emphasizing its tension-reducing effects. All patients who presented for axillary reconstruction after radical excision of chronic inflammatory skin lesions between May 2019 and December 2020 were identified using the medical record database. Eleven patients ranging in age from 17 to 71 years underwent modified KPIF axillary reconstruction. Four types of modifications (modified type II KPIF, omega variation closure, Sydney melanoma unit modification, and hemi-KPIF) were used. All defects (size range, 2.5 × 3 cm^2^ to 8 × 13 cm^2^) were successfully covered using these modified KPIF techniques. All flaps (size range, 3.5 × 3.5 cm^2^ to 11 × 30 cm^2^) fully survived without complications. All patients exhibited favorable functional outcomes, and no cases of recurrence or limitations in joint range of motion were observed during the follow-up period (range, 4–5 months). Modified KPIF techniques may represent a reliable, effective alternative reconstructive modality for managing axillary defects.

## 1. Introduction

Reconstruction of axillary defects resulting from radical excision of chronic inflammatory skin lesions, such as complicated epidermoid cysts (ECs) and hidradenitis suppurativa (HS), is often challenging. The axilla region, which is also known as the axillary pouch, contains several hair follicles and sebaceous glands, and it is densely packed with eccrine and apocrine sweat glands [1]. Therefore, the axilla is a common site for ECs and HS [2, 3], which frequently progress to chronic inflammatory skin lesions. Surgical treatments for these lesions generally include radical resection, which leads to skin and soft tissue defects of various sizes [2, 4].

There are various reconstructive options for covering the axillary defect, including direct closure with or without undermining, healing via secondary intention, dermal substitutes, skin grafts, local flaps, and free flaps [5, 6]. Reconstructive surgeons select the appropriate option for covering the axillary defect based on its size and depth. Small axillary defects can be reconstructed either via direct closure with undermining or healing with secondary intention; however, moderate defects should be covered with local flaps, and large or extensive defects should be covered with free flaps [5]. In terms of the depth of the defect, skin grafts can be applied for superficial defects, and flap techniques are useful for covering deep defects involving exposure of the underlying structures [7].

Several local flaps have been developed to cover axillary defects [8–10]. There is no single remarkable local flap technique for axillary reconstruction. Previous studies have demonstrated that axillary reconstruction with a pedicled perforator flap, such as a thoracodorsal artery perforator flap improves quality of life, shortens the healing period, and decreases the risk of complications [11]. Among the numerous types of local flaps, the keystone perforator island flap (KPIF) technique devised by Behan in 2003 has demonstrated successful outcomes in a series of 300 cases. This reconstructive modality can be applied to cover various defects in most parts of the human body and is used by many reconstructive surgeons as a primary option or an alternative to other reconstructive methods [4, 5, 7, 12, 13]. The popularity of the KPIF technique is thought to be attributable to its distinct differences from other flaps, including its intuitive defect-adjustable design and easy reproducibility; even beginners can perform this technique with a minimal learning curve [4, 5, 7, 13]. Several studies have reported the use of the KPIF for reconstructing axillary defects [2, 14]; however, relatively more studies have compared KPIF reconstruction in other body areas. This report presents our experience with KPIF reconstruction for the coverage of axillary defects secondary to radical excision of chronic inflammatory skin lesions. Our experience may help to expand the versatility of KPIF reconstruction in the field of plastic surgery by demonstrating the utility of KPIF reconstruction in the axillary region.

## 2. Materials and Methods

The study protocol and its research procedures conformed to the ethical guidelines of the 1975 Declaration of Helsinki. The Ethical Review Board of Konyang University Hospital approved this work (approval number: 2020-08-016). We obtained written informed consent from all patients to publish the information and images in an online open-access publication.

We performed a retrospective review of data for patients who underwent KPIF reconstruction to cover axillary defects secondary to radical excision of chronic inflammatory skin lesions between May 2019 and December 2020. Patients who underwent only KPIF reconstruction of the axilla were included; however, those who underwent axillary reconstruction using the KPIF technique combined with other local flap techniques were excluded.

### 2.1. Surgical Techniques

Routine preoperative wound preparations were used to control surrounding infections and promote perfusion around the wounds of chronic inflammatory skin lesions of the axilla, including empirical antibiotic treatment and wound dressing for at least 1 to 2 weeks [4, 5, 13, 15]. Our empirical antibiotic regimen included intravenous injection of either cefazedone sodium 1 g or amoxicillin sodium 1.2 g. Our wound dressing protocol included conventional dressing with foam and packing materials to control acute inflammation, followed by negative pressure wound therapy to promote tissue perfusion and stabilization. After achieving adequate wound preparation and stabilization through these management procedures, we performed radical excision followed by KPIF reconstruction [4, 5, 13, 15].

**Figure 1:**
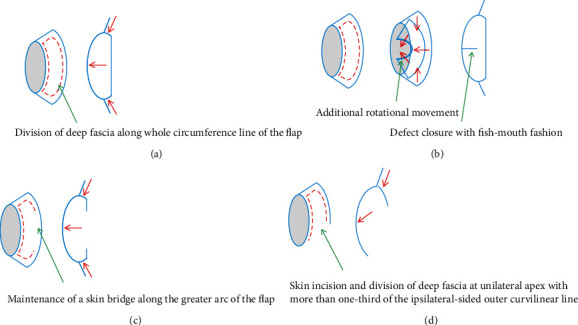
Basic illustration of the four modifications of the keystone perforator island flap (KPIF) used in this study. (a) Modified type II KPIF. (b) Omega variation closure (OVC) KPIF. (c) Sydney melanoma unit modification (SMUM) KPIF. (d) Hemi-KPIF. Red dotted lines represent division of deep fascia, and red arrows represent direction of flap movement.

All operative procedures were performed under general anesthesia by the senior author. First, radical excision was performed, which included complete excision of the lesion (such as cystic mass components, sinus tracts, fistulas, and abscess pockets), debridement of the surrounding unhealthy tissues, and release of the surrounding adhesions [4]. Then, the final defect was created. The KPIF was designed based on the final defect size, surrounding tissue laxity, and relaxed skin tension lines (RSTLs) of the axilla [4, 5, 7, 13]. We designed the KPIF to have a larger width than the defect at the side with sufficient tissue laxity and attempted to create the KPIF long axis, so that it was as parallel to the RSTLs as possible [4, 5, 7, 13]. We used several modifications of the KPIF, including the modified type II KPIF [13], omega variation closure (OVC) KPIF [16], Sydney melanoma unit modification (SMUM) KPIF [17], and hemi-KPIF [18].  Figure 1 shows these KPIF modifications.

The original four subtypes of the KPIF devised by Behan are type I (skin incision only), type II (A: division of the deep fascia along the outer curvilinear line; B: division of the deep fascia and skin graft secondary to the defect), type III (double-opposing KPIFs), and type IV (KPIF with undermining of up to 50% of the subfascial flap) [12]. Compared with the original type IIA KPIF, the modified type II KPIF used for this study had the following characteristics. The division of the deep fascia was limited to the area along the outer curvilinear line of the original type IIA KPIF, but it included the whole circumference line of the flap of the modified type II KPIF [13]. The OVC KPIF includes additional rotation of the flap, which results in a fish mouth type of closure [16]. The SMUM KPIF entails the maintenance of a skin bridge along the outer curvilinear line [17]. The hemi-KPIF involves a unilateral incision of the curvilinear portion of the flap as necessary until the defect is closed [18]. We decided which modification to use intraoperatively instead of preoperatively [19]. We did not routinely use an intraoperative Doppler device to locate the hot spot of the skin perforators because we considered the axilla to be a perforator-rich area. A skin incision with dissection from the subcutaneous layer to the deep fascia was initially performed at the unilateral apex and involved more than one-third of the ipsilateral-side outer curvilinear line, which created the hemi-KPIF. If the defect was not covered by the hemi-KPIF alone, we then performed another skin incision with dissection from the subcutaneous layer to the deep fascia at the other apex and maintained a skin bridge along the outer curvilinear line, which created the SMUM KPIF. If we considered the flap vascularity to be sufficient and stable, then the remaining skin bridge was incised and dissected to create the type II KPIF. Then, we performed minimal undermining at the flap margin, which contributed to preserving vascularity through the central hot spot of perforators [4, 5, 7, 13]. After achieving fastidious bleeding control, the insetting of the flap was performed using the following sequence: first, the defect side of the flap was sutured using either linear closure or OVC in case further flap movement was necessary to reduce closure tension; next, V-Y advancement closure at either the unilateral apex (in the case of the hemi-KPIF) or bilateral apexes (in the case of other modifications) of the flap was performed; finally, the donor site of the flap was sutured. At the end of surgery, a mild compressive foam dressing was applied.

We retrospectively reviewed each patient's electronic medical chart, including the defect cause, defect and flap sizes, type of KPIF, postoperative complications, flap survival, follow-up durations, and outcomes such as scar appearance and joint range of motion (ROM). ROM in the glenohumeral joint of the affected side was evaluated and compared with that of the unaffected side in each case during the final follow-up by the senior author.

## 3. Results

Eleven patients (9 male and 2 female) with an average age of 41.73 years (±17.38 years; range, 17–71 years) were included.  Table 1 summarizes the patient characteristics and clinical data. Two patients had comorbidities (diabetes mellitus and hypertension in case 4 and hypertension in case 10). Lesions were unilateral in all cases. The defect causes included radical excision of complicated ECs with surrounding cellulitis in eight patients and radical excision of HS in the remaining three patients. The defect sizes ranged from 2.5 × 3 cm^2^ to 8 × 13 cm^2^, and all defects were successfully covered with the modified KPIF technique. The flap sizes ranged from 3.5 × 3.5 cm^2^ to 11 × 30 cm^2^. The following types of KPIF were used: hemi-KPIF, two cases; SMUM KPIF with OVC, seven cases; modified type II KPIF, two cases. All flaps completely survived without flap-related complications. No postoperative complications such as wound infection, dehiscence, hematoma, or seroma occurred. The final outcomes at an average of 4.36 months (±0.50 months; range, 4–5 months) after surgery were linear scar formation (7 cases) and hypertrophic scar formation (4 patients). No joint ROM limitations occurred on the affected side, including shoulder extension (0–60°), flexion (0–180°), and abduction (0–180°).

### 3.1. Case Presentations

#### 3.1.1. Case 1

**Figure 2:**
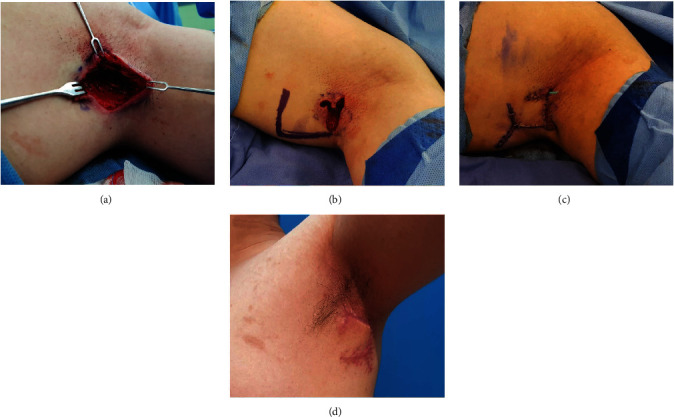
Clinical photographs (case 1). (a) Final defect (3 × 3.5 cm^2^) after radical excision of the complicated epidermoid cyst (EC) in the left axillary pouch. (b) Design of the hemi-keystone perforator island flap (KPIF) (4 × 4 cm^2^) on the medial side of the defect. (c) Successful coverage of the defect using the hemi-KPIF. (d) Postoperative photograph after 4 months of follow-up showing no EC recurrence and a hypertrophic scar.

A 30-year-old man presented with an approximately 2 cm solid lesion with an indefinite border and surrounding inflammation in the left axillary pouch (Figure 2). We diagnosed his lesions as complicated ECs with surrounding cellulitis and planned surgical management. Before surgery, conventional dressings and empirical antibiotic treatments were applied for 1 week for wound preparation. Then, we performed radical excision followed by modified KPIF reconstruction. The final defect size was 3 × 3.5 cm^2^. We covered the defect with a hemi-KPIF (4 × 4 cm^2^) from the medial side of the defect. The inset of the flap and closure of the donor site were performed without tension. The flap fully survived without postoperative complications. The patient did not experience EC recurrence and achieved a favorable functional outcome without limitations in joint ROM during 4 months of follow-up.

#### 3.1.2. Case 5

**Figure 3:**
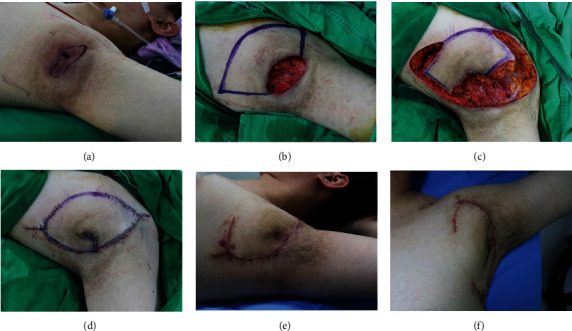
Clinical photographs (case 5). (a) A chronic inflammatory skin lesion was diagnosed as hidradenitis suppurativa (HS) in the left axillary pouch. (b) Final defect (6 × 8 cm^2^) after radical excision of the lesion and design of a keystone perforator island flap (KPIF) (8 × 19 cm^2^) on the medial side of the defect. (c–d) Successful coverage of the defect using the Sydney melanoma unit modification KPIF with omega variation closure. (e–f) Postoperative photograph after 4 months of follow-up shows no HS recurrence and a hypertrophic scar.

A 22-year-old man had a 2-year history of a recurrent inflammatory skin lesion in the left axillary pouch (Figure 3). Central abscess formation with surrounding inflammation and broad surrounding scarring were observed during the physical examination. We diagnosed his lesion as HS (Hurley grade II) and planned surgical management. Before surgery, conventional dressings and empirical antibiotic treatments were applied for 2 weeks for wound preparation. Then, we performed radical excision followed by modified KPIF reconstruction. The final defect size was 6 × 8 cm^2^. We covered the defect with a SMUM KPIF with OVC (8 × 19 cm^2^) from the medial side of the defect. The inset of the flap and closure of the donor site were performed without tension. The flap fully survived without postoperative complications. The patient did not experience HS recurrence and achieved a favorable functional outcome without limitations in joint ROM during 4 months of follow-up.

#### 3.1.3. Case 8

**Figure 4:**
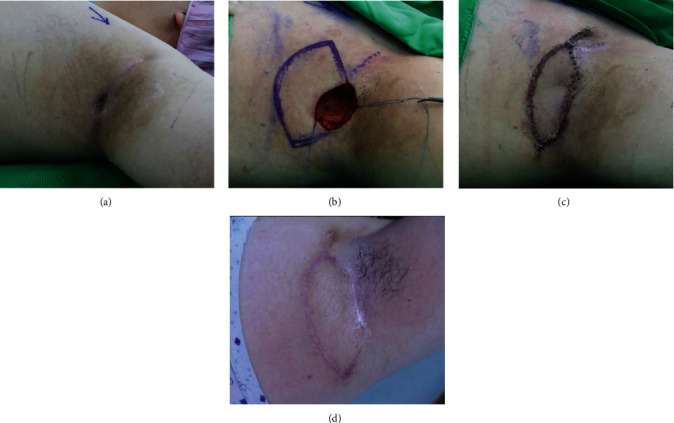
Clinical photographs (case 8). (a) A chronic inflammatory skin lesion was diagnosed as a complicated epidermoid cyst (EC) in the left axillary pouch. (b) Final defect (3 × 4 cm^2^) after radical excision of the lesion and design of a keystone perforator island flap (KPIF) (3.5 × 8.5 cm^2^) on the medial side of the defect. (c) Successful coverage of the defect using the modified type II KPIF. (d) Postoperative photograph after 5 months of follow-up shows no EC recurrence and a linear scar.

A 17-year-old boy presented with an approximately 2.5 cm solid lesion with an indefinite border and surrounding inflammation in the left axillary pouch (Figure 4). We diagnosed his lesions as complicated ECs with surrounding cellulitis and planned surgical management. Before surgery, we administered conventional dressings and empirical antibiotic treatments for 1 week for wound preparation. Then, we performed radical excision followed by modified KPIF reconstruction. The 3 × 4 cm^2^ final defect was covered with a 3.5 × 8.5 cm^2^ modified type II KPIF from the medial side of the defect. Full flap survival was achieved without postoperative complications. No EC recurrence occurred, and the patient experienced a favorable functional outcome with no limitations in joint ROM during 5 months of follow-up. Supplement 1 shows the postoperative shoulder joint movement at the 5-month follow-up evaluation.

## 4. Discussion

The present report describes a single surgeon's experience with KPIF reconstruction in 11 consecutive cases of axillary defect secondary to radical excision of chronic inflammatory skin lesions. We attribute the favorable outcomes with complete flap survival for all cases to sufficient preoperative wound preparation, complete excision of the lesions, and adequate application of the KPIF modifications.

In terms of the general reconstructive principle, any defects with surrounding inflammatory tissues must be approached carefully from the perspective of local flap reconstruction. Under the zone of injury, such as an inflammatory wound bed, the flap is vulnerable to reduced tissue laxity and decreased vascular perfusion [5, 15]. Additionally, wound healing problems and complications can occur in this zone of injury [5, 15]. All defects reported here developed after radical excision of a chronic inflammatory skin lesion in the axilla. Preoperative wound preparations, including empirical antibiotic treatment and wound dressing, were applied in all patients for at least 1 to 2 weeks. Radical excision of the lesion followed by adequate local coverage is crucial to successful reconstruction and prevention of the recurrence of chronic inflammatory skin lesions [2]. For all our cases, flap coverage was performed after complete excision of the lesion with surrounding debridement, and adhesion release was achieved. We consider that this management strategy resulted in complete flap survival without wound healing complications, surgical site infections, or recurrences.

The axillary pouch is a complex three-dimensional area that comprises thin skin and pliable soft tissue that allow intact shoulder movement through the motion of the glenohumeral joint [20]. Therefore, it is crucial to achieve tension-reducing wound closure when reconstructing the axillary defect, which can prevent scar contracture and maintain glenohumeral joint movement [5]. Additionally, it is necessary to provide thin, flexible, and durable tissues during the reconstruction of the axillary defect to bear continuous motion with multivector tensile and shearing forces [5, 21]. These characteristic features of the axillary region may complicate wound closure or lead to wound healing problems, making it difficult to cover even small defects. Therefore, during axillary reconstruction, reconstructive surgeons should consider the tension induced by shoulder movement, which can be high in the sutured area after wound closure and covering the defect with similar thin and flexible tissues. The goals of local flap reconstruction in the axilla are covering the defect through the replacement of insufficient tissues and achieving problem-free wound healing by reducing wound tension at the coverage area.

The KPIF technique involves looking at the defect through two opposing V-Y advancement flaps, similar to the keystone of Roman arches [12, 13]. Behan elucidated that the movements of the V-Y advancement flap of either apex result in surrounding tissue laxity, redistribute wound tension perpendicular to the direction of each flap movement, and eventually reduce wound closure tension [12, 13, 22, 23]. Several researchers have argued that the KPIF technique does not achieve tension reduction [24]. However, the tension-reducing effect of the KPIF has been verified by two *in vivo* studies [4, 13]. During those previous studies, the KPIF movement resulted from the stepwise tissue layer release that involved the skin, subcutaneous layer, and deep fascia layer at the time of flap dissection [4, 13]. Moreover, the KPIF technique has a greater tension-reducing effect than releasing other layers for division of the deep fascia [13]. We used four modifications of the KPIF that involved the division of the deep fascia. Wound closure with minimal tension is essential for axillary reconstruction. We were able to achieve favorable results using these KPIF modifications. Furthermore, there were no limitations in joint ROM or shoulder movement in our patients, suggesting that the tension-reducing effect of the KPIF can allow the axillary defect to effectively bear changes in tensile force created by joint movement.

**Figure 5:**
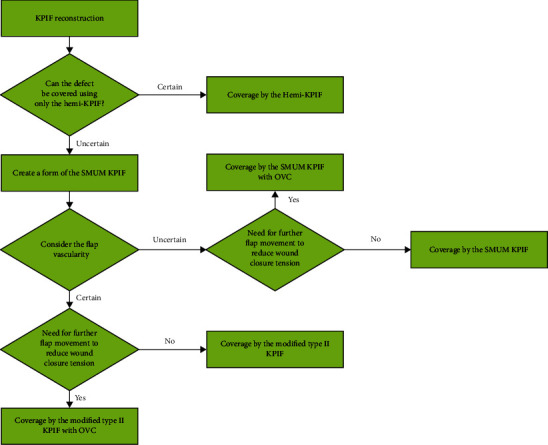
An algorithm for the stepwise application of the modified keystone perforator island flap (KPIF) technique. SMUM, Sydney melanoma unit modification; OVC, omega variation closure.

The modified type II KPIF can remarkably reduce tension and improve movement when compared with other modifications because of the complete division of all surrounding tissue layers, similar to the true island flap [13]. The OVC KPIF allows additional rotation of the flap and can further reduce closure tension via rotational flap movement [16, 19]. The SMUM KPIF, introduced by Moncrieff et al. in 2008, can provide additional vascularity and structural stabilization of the flap by maintaining a skin bridge along the outer curvilinear line of the KPIF [17, 19]. Finally, the hemi-KPIF, developed by Petukhova et al. in 2020, is an economical modification of the KPIF because of its minimized incision area, decreased morbidity, and increased efficiency. Furthermore, it can be especially useful for the closure of areas that are expected to have tension with primary closure or direct closure difficulties [18]. In the current series, SMUM KPIF and OVC were used together in all cases. We consider this combination very useful because the SMUM KPIF secures flap stability, while OVC allows further flap movement. We achieved full flap survival without flap-related complications in all cases through the appropriate application of these four modifications. Based on our experience, we developed an algorithm for the stepwise application of the modified KPIF (Figure 5). This algorithm may enable more efficiently and economic determination of the appropriate KPIF technique in clinical practice based on the characteristics of each modification. When applying this algorithm, the flap movement for each type of defect coverage and flap vascularity should be considered at each step. Although not used in the present study, a Doppler device or an indocyanine green fluorescence device may aid in the assessment of flap vascularity.

Despite the success of axillary reconstruction and favorable outcomes, our research had some limitations. First, the sample size was comparatively small, and the study design was neither prospective nor randomized. Furthermore, this retrospective case series did not include a comparison group, which may have inadvertently led to selection and confounding bias. Therefore, we have planned additional studies with a prospective, large-scale design, and a comparison group to ensure the validity of the consistent outcomes observed for axilla reconstruction using modified KPIFs. Second, additional incision scars and abnormal scars, such as hypertrophic scars and keloids, can be problematic in some cases. Hypertrophic scars developed in four of our patients, which were managed using triamcinolone injections. Therefore, the possibility of scarring and appropriate strategies for postoperative scar management should be explained to patients before surgery. Third, the follow-up period for our cases was relatively short. Postoperative scar formation can require 12 months or more; however, the average follow-up period among the current cases was 4.36 ± 0.50 months. Future studies with longer follow-up periods are essential for determining definitive outcomes. Finally, although the modified KPIF can be a reliable option for covering axillary defects, it is not the only available option. Previous studies have suggested several local flap techniques as good reconstructive methods for axillary defects [25–27]. The posterior arm flap technique is a simple and reliable method that can achieve a brachioplasty effect [25]. The pedicled thoracodorsal artery perforator flap technique is a versatile reconstructive option because it preserves arm abduction in cases of severe axillary HS [26]. In addition, the freestyle perforator puzzle flap technique is also a valid method for axillary reconstruction in patients with large and complicated defects [27]. Therefore, the modified KPIF technique is not always necessary for axillary reconstruction. Rather, the reconstructive modality considered most suitable for each case should be applied.

## 5. Conclusions

The present study conformed to the Strengthening the Reporting of Observational studies in Epidemiology (STROBE) guidelines as an observational study (Supplement 2). We examined different KPIF reconstruction techniques for axillary defects secondary to radical excision of chronic inflammatory skin lesions. Based on our successful results, we believe that modified KPIF techniques (modified type II KPIF, OVC, SMUM, and hemi-KPIF) are good alternatives to other methods for covering axillary defects that can achieve reliable reconstruction by reducing tension and replacing like tissue with like tissue. Future studies will be performed to objectively evaluate the tension-reducing effect of axillary KPIF reconstruction and solidify the present outcomes.

## Figures and Tables

**Table 1:** Patient data.

Case	Sex/age	Defect cause	Defect size (cm^2^)	Flap size (cm^2^)	Type of KPIF	Flap survival	Complications	Follow-up duration (months)	Limitation of joint ROM (affected side)	Final outcome
1	M/30	Complicated EC with surrounding cellulitis	3 × 3.5	4 × 4	Hemi-KPIF	Fully survived	None	4	None	Hypertrophic scar
2	M/58	Complicated EC with surrounding cellulitis	4 × 5	7.5 × 14	SMUM KPIF with OVC	Fully survived	None	5	None	Linear scar
3	M/42	Complicated EC with surrounding cellulitis	3 × 3.5	4 × 4	Hemi-KPIF	Fully survived	None	4	None	Linear scar
4	M/71	Complicated EC with surrounding cellulitis	3.5 × 4	5 × 12	SMUM KPIF with OVC	Fully survived	None	4	None	Linear scar
5	M/22	Hidradenitis suppurativa	6 × 8	8 × 19	SMUM KPIF with OVC	Fully survived	None	4	None	Hypertrophic scar
6	F/34	Complicated EC with surrounding cellulitis	2.5 × 3.5	3.5 × 6	Modified type II KPIF	Fully survived	None	4	None	Linear scar
7	M/51	Hidradenitis suppurativa	8 × 13	11 × 30	SMUM KPIF with OVC	Fully survived	None	5	None	Hypertrophic scar
8	M/17	Complicated EC with surrounding cellulitis	3 × 4	3.5 × 8.5	Modified type II KPIF	Fully survived	None	5	None	Linear scar
9	M/29	Hidradenitis suppurativa	4.5 × 5.5	7 × 13	SMUM KPIF with OVC	Fully survived	None	4	None	Hypertrophic scar
10	F/42	Complicated EC with surrounding cellulitis	2.5 × 4	3.5 × 6.5	SMUM KPIF with OVC	Fully survived	None	4	None	Linear scar
11	M/63	Complicated EC with surrounding cellulitis	2.5 × 3	3 × 5	SMUM KPIF with OVC	Fully survived	None	5	None	Linear scar

M, male; F, female; EC, epidermoid cyst; KPIF, keystone perforator island flap; OVC, omega variation closure; SMUM, Sydney melanoma unit modification; ROM, range of motion.

## Data Availability

The data presented in this study are available on request from the corresponding author (K.N.K.). The data are not publicly available due to privacy restrictions.

## Supplementary Materials

Supplementary Materials Supplement 1: shows postoperative shoulder joint movement of a patient (case 8) at the 5-month follow-up evaluation. The STROBE checklist is also provided as Supplement 2.
