# Supplementary material for: Modified Keystone Perforator Island Flap for Tension-Reducing Coverage of Axillary Defects Secondary to Radical Excision of Chronic Inflammatory Skin Lesions: A Retrospective Case Series
Source: Biomed Res Int. 2022 Sep 29;2022:5600450. doi: 10.1155/2022/5600450 (PMC9537010; doi:10.1155/2022/5600450)
Supplement: Supplementary Materials — Supplement 1: shows postoperative shoulder joint movement of a patient (case 8) at the 5-month follow-up evaluation. The STROBE checklist is also provided as Supplement 2. [file 5600450.f1.zip › Supplementary Material.docx]

Supplementary Material

Supplementary Video can be downloaded from <https://editage.sharefile.com/d-s8312f7410ed0414ba2c769fe359fe6f9>
